# Current Knowledge on the Interaction of Human Cytomegalovirus Infection, Encoded miRNAs, and Acute Aortic Syndrome

**DOI:** 10.3390/v15102027

**Published:** 2023-09-29

**Authors:** Francesco Nappi, Almothana Alzamil, Sanjeet Singh Avtaar Singh, Cristiano Spadaccio, Nicolas Bonnet

**Affiliations:** 1 Department of Cardiac Surgery, Centre Cardiologique du Nord, 93200 Saint-Denis, France; almothana.md@gmail.com (A.A.); drnicolasbonnet@gmail.com (N.B.); 2 Department of Cardiothoracic Surgery, Royal Infirmary of Edinburgh, Edinburgh EH16 4SA, UK; sanjeet.singh@glasgow.ac.uk; 3 Department of Cardiothoracic Surgery, Mayo Clinic, Rochester, Rochester, MN 55905, USA; cristianospadaccio@gmail.com

**Keywords:** human cytomegalovirus, miRNAs, acute aortic syndrome

## Abstract

Aortic dissection is a clinicopathological entity caused by rupture of the intima, leading to a high mortality if not treated. Over time, diagnostic and investigative methods, antihypertensive therapy, and early referrals have resulted in improved outcomes according to registry data. Some data have also emerged from recent studies suggesting a link between Human Cytomegalovirus (HCMV) infection and aortic dissection. Furthermore, the use of microRNAs has also become increasingly widespread in the literature. These have been noted to play a role in aortic dissections with elevated levels noted in studies as early as 2017. This review aims to provide a broad and holistic overview of the role of miRNAs, while studying the role of HCMV infection in the context of aortic dissections. The roles of long non-coding RNAs, circular RNAs, and microRNAs are explored to identify changes in expression during aortic dissections. The use of such biomarkers may one day be translated into clinical practice to allow early detection and prognostication of outcomes and drive preventative and therapeutic options in the future.

## 1. Introduction

Acute aortic syndrome (AAS) is a clinical and pathological entity promoted by the rupture of the intima of the aorta due to pathological factors. Approximately 90% of patients with AAS have an aortic dissection (AD) while an intramural hematoma develops in the remainder [1]. Aortic dissection, albeit an uncommon disease, clinically occurs suddenly and confers a high mortality [2,3,4,5]. The most accepted classification in the field of aortic pathology divides aortic dissection into Stanford type A (TAAD) and Stanford type B (TBAD). The former affects the ascending aorta rather than the latter [1]. In both Stanford types of aortic dissection, the blood enters the media through a rupture point, causing a section of the media into two layers, internal and external, to form a false lumen. Under the thrust exerted by pressure, the dissection in the aortic wall enlarges, generating a voluminous parietal thrombus that can stop the progression toward the complete rupture of the aorta [6].

Given the continuous development of diagnostic investigative methods, surgical procedures dictating immediate treatment, and optimal guideline-directed medical therapy based on antihypertensive therapy, the mortality rate of TAAD is decreasing as suggested in several reports of international registries [7,8,9,10,11,12]. However, there remain concerns regarding the high mortality and morbidity. First, although the typical symptom of AAS is the sudden onset of pain that may drive the correct diagnosis [13,14], an erroneous assessment of AAS may occur in some patients with an initial clinical presentation characterized by the appearance of atypical symptoms attributed to infarction of the myocardium, atypical abdominal pain, stroke, and systemic malperfusion. Secondly, the current clinical diagnosis of AAS appears to be insufficient through the standard diagnostic methods, including computed tomography, transthoracic echocardiography, transesophageal echocardiography, and magnetic resonance imaging, which have some limitations [15,16]. For these reasons, it is urgently desirable to achieve a better understanding of the molecular processes underlying the pathological damage that sustains TAAD, to undertake more effective diagnostic strategies that identify critical molecules to be detected early. Therefore, the main route that leads to the early and rapid diagnosis of AAS must exploit newer methods that allow it to identify concomitant causes and clarify the pathological mechanism of this disease at the same time. Ultimately, an early diagnosis and appropriate therapeutic choices may be more effective in reducing mortality.

Based on the substantial importance currently attributed to the role of miRNAs in the diagnosis and therapy of many cardiovascular diseases [17], as well as their expression during viral infection [18], we investigated whether HCMV-encoded miRNAs in plasma could be employed as a safe, stable, and specific biomarker for AAS monitoring. We also evaluated the emerging role of long non-coding RNAs and circular RNAs in AAS and whether there is a correlation with HCMV infection. 

## 2. Pathophysiology

**Figure 1:**
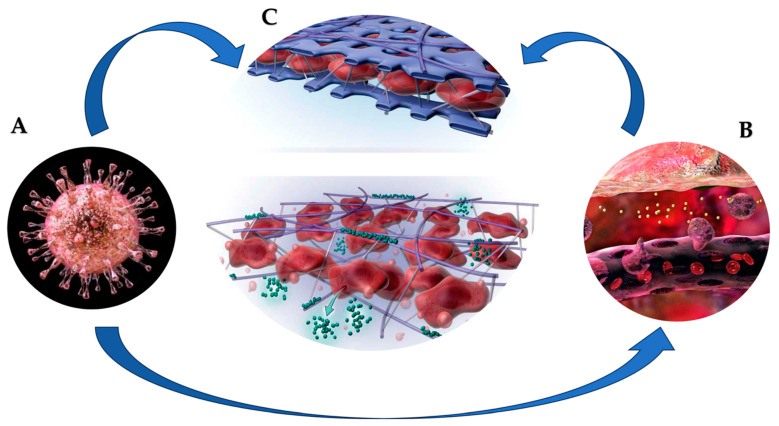
(**A**). HCMV is stored in aortic fibroblasts, which are involved in differentiation and production of collagen and elastin fibrils. HCMV can elicit latent chronic inflammation of vessel wall (**B**). In these cases, a disorder in the structural support and elasticity of the aorta leads to the disintegration of the alternating layers of elastic lamellae and smooth muscle cells. (**C-up**). In patients with AAS, the structural support and elasticity of aorta characterized by alternating layers of elastic lamellae (blue) and smooth-muscle cells (dark red) is damaged. (**C-down**). The structural alteration of fibrillin 1 secondary to inflammatory triggers can promote rupture at different levels of the aortic wall. A series of events can lead to an aorta with weakened structural integrity and reduced elasticity. Consumption of this glue protein culminates in a disrupted layer architecture whereby smooth muscle cells slough off, accompanied by increased local levels of matrix metalloproteinases (green/MMPs), leading to loss of integrity in the extracellular matrix and accumulation of apoptotic cells [19,20].

The aorta has a complex structure with peculiar morphological characteristics. HCMV is stored in aortic fibroblasts, which constitute the most abundant cell line disseminated in the connective tissue of the vasculature [19]. Fibroblasts work to provide the production and differentiation toward the production of fibrils of collagen and elastin. Structural support and elasticity of the aorta are afforded by means of alternating layers of elastic lamellae and smooth-muscle cells. At the histologic level, the smooth-muscle cells in the aorta in persons with the normal structure of vessel walls are secured to the adjacent elastin and collagen matrix by fibrillin 1 microfibrils. In patients with AAS, the aorta may develop a disruption at the level of different layers mediated by fibrillin 1 structural alteration secondary to inflammatory triggers. This process promotes the consumption of this glue protein that culminates in a disrupted architecture whereby smooth-muscle cells detach, accompanied by a surge in local levels of matrix metalloproteinases (MMPs), leading to loss of integrity in the extracellular matrix and the accumulation of apoptotic cells. These events may lead to an aorta with weakened structural integrity and reduced elasticity (Figure 1) [20].

Molecular biology has pointed out how the key damage of type A aortic dissection (TAAD) is correlated with the disturbance of cell adhesion, degradation of the extracellular matrix (ECM), and related inflammation promoted by injury against endothelial cells and vascular smooth muscle cells (VSMCs) [21,22]. It has also been observed that these mechanisms are associated with other biological processes that support hypoxia and the change in the phenotype of smooth muscle cells [23,24]. Although advances in biomolecular research have been considerable, the senior regulatory mechanisms that orchestrate this multi-event disease process remain uncertain. A regulatory function is attributed to microRNAs (miRNAs).

The pivotal study pertaining to the regulation mechanism of miRNA in individuals experiencing aortic acute syndrome dates back to 2017 [25]. However, the protocol mandated the assessment of findings both on aortic dissection and aortic aneurysm. Yu and colleagues studied aortic tissue recovered from patients with AAS and aortic aneurysm. The investigators observed that miR-30a was markedly overexpressed in contrast to lysyl oxidase (LOX) and elastin, which were significantly lower in the examined patients. The discovery of luciferase subsequently confirmed the identification that miR-30a binds to LOX in a targeted manner, further enhancing the regulation of LOX expression within VSMCs. Finally, evidence resulted from animal experiments with additional confirmation that inhibition of miR-30a expression inhibited the onset of aortic dissection in mice [25]. This crucial human study on miRNA targets was addressed to the aortic dissection followed by other animal studies that validated these findings. In nearly every subsequent investigation, the method used to explore the relationship between miRNA and aortic dissection was emphasized and assessed based on changes in cell phenotype or on the regulation of signaling pathways, thereby allowing the reveal of regulatory mechanisms exerted by miRNAs. For instance, Sun and colleagues illustrated how miR-27a controls vascular reconstruction through apoptosis of endothelial cells and interactions with VSMCs. Subsequently, further studies in mice reported that the occurrence of aortic dissection was inhibited by overexpression of miR-27 [26].

In the study by Wang and colleagues, miR-134-5p was shown to exert substantial inhibition in the phenotypic transformation and migration of VSMCs. The mechanism was based on the interaction with the STAT5B/ITGB1 gene, which regulates aortic dilatation and medial vascular degeneration. The crucial finding that emerged was the inhibition of aortic dissection in mice in which miR-134-5p was overexpressed [27]. Moreover, the knowledge of the interconnection mechanisms between miRNA and dissection identified as an upstream factor in promoting AD can be used as an intermediate factor to prevent AD. The role of an intermediate factor emerges in the action of adiponectin. In fact, Duan and colleagues showed that miR-133a is upregulated by adiponectin with an effect on the inhibition of the pyroptosis pathway of cells. A series of inflammatory factors such as caspase-1, interleukin-1 β (IL-1 β), IL-18, and osteopontin (OPN) are inhibited, resulting in aortic dissection [28].

Xu and colleagues in patients with TAAAD extracted long noncoding RNA (lncRNA) and found both 393 lncRNAs and 432 mRNAs to be abnormally expressed. Among them, TNFSF14 was negatively correlated with MMP14 and MMP19 with a Pearson correlation coefficient that ranged from −7.0 to 8.5. Finally, lnc-TNSF14 may play a key role in regulating matrix degradation, which may influence the development of type B aortic dissection [29].

## 3. HCMV, RNAs, and Aortic Dissection

**Figure 2:**
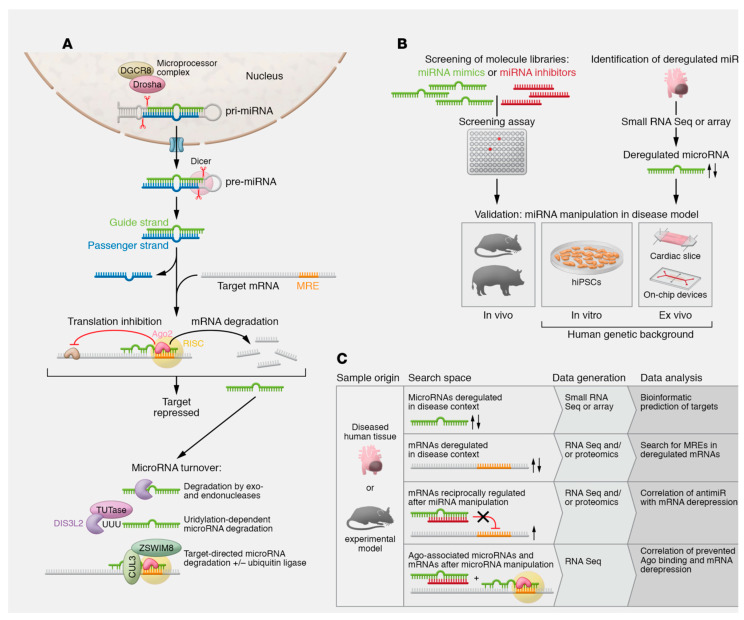
The biogenesis and function of microRNA (miRNA) are depicted. (**A**) The mechanism of action and degradation pathways of microRNAs are also significant factors to consider. Canonical microRNA biogenesis involves larger hairpin RNA molecules called pri-miRNAs. These pri-miRNAs arise from RNA Pol II transcription of microRNA genes or clusters or are found within introns. A microprocessor complex, consisting of the endonuclease Drosha, the DiGeorge critical region 8 protein (DGCR8), and other factors, cleaves these pri-miRNAs, activating their functions. The pre-miRNA produced is transported to the cytoplasm and fragmented by the enzyme Dicer. It is tailored to a length of 21 to 22 nucleotides. Occasionally, certain non-canonical mechanisms of microRNA formation bypass the Dicer or microprocessor complex. Afterwards, one of the resulting duplexes, made up of 21–22 nucleotides, is incorporated into the RNA-induced silencing complex (RISC) as the guide strand. Meanwhile, the redundant passenger strand or *-strand undergoes prompt degradation. If both strands are maintained, they can take on individual functions, as shown for cardiovascular miR-21 and miR-126. There are also exceptions where microRNA strands localize to the nucleus and function in unconventional ways. The degradation of microRNAs involves exonucleases XRN-1, PNPase old-35, and RRP41 or the endonuclease Tudor-SN. The nuclease DIS3L2 degrades a subset of microRNAs after modification by terminal uridyltransferases (TUTases). Target-directed microRNA degradation mechanisms have been established, which includes the participation of ubiquitin ligases. (**B**) Strategies for determining and validating cardiovascular microRNAs relevant to diseases. (**C**) Methods for identifying microRNA targets. Refs. [34,35,36,37,38,39]. From Laggerbauer B et al. J Clin Invest. 2022 Jun 1;132(11): e159179. ∗ Arrow; increase or decrease.

Small RNAs act as regulators as they are mostly present in plasma, saliva, and other bodily fluids [30]. The action of miRNAs is essentially to promote an inhibition in the translation of related proteins. This work is accomplished by integrating a site in the 3′-untranslated region (3′ UTR) of its targeted mRNA to degrade the mRNA or block the mRNA translation machinery [31]. The direct consequence of this action offers the explanation of the role of miRNAs as actors of many biological processes in which they are implicated in gene regulation to encode proteins. Therefore, miRNAs are associated with many allergic diseases, cardiovascular disease, cancer, and acute aortic syndrome [17,32,33]. Again, we learned that although individual miRNAs lack a marked disease specificity, crucial miRNA combinations have been observed to be associated with certain diseases [34]. It was observed that changes resulting in miRNA expression are of paramount importance to reach the diagnosis and treatment of diseases [35]. Moreover, several studies have suggested that a variety of miRNAs derived from host and pathogenic microbes are highly stable in plasma and serum, and that plasma and serum alterations of these circulating miRNA levels may serve as noninvasive biomarkers for the discovery of various diseases (Figure 2) [36,37,38,39].

Human cytomegalovirus (HCMV) is included in the herpes virus family and, of this family, HCMV has the largest genome that can encode more than 200 types of proteins [40]. The HCMV infection rate is very high, reaching 40% to 99% of the population who have been in contact with this virus [41]. Although individuals with a competent immune response can avoid the appearance of symptoms following the infection, the same does not occur in newborn populations and immunosuppressed persons who, once infected with HCMV, can develop clinical symptoms [42]. We are aware of the fundamental molecular mechanism by which HCMV-encoded miRNAs promote coexistence between the virus and host cells or host cell lysis. Thus, HCMV through miRNA expression may control the host response through a precise regulatory mechanism [43]. In the field of molecular research, a fundamental role has been observed for 26 mature HCMV-induced miRNAs [44]. Under the immune-inflammatory profile, HCMV-miRNAs promote an inhibitory effect on the function of natural killer cells (NK cells) and cytotoxic T cells, as well as a crucial action in the suppression of the inflammatory response that works in concert with the immune response, thereby causing a complicated dysregulation of the immuno-inflammatory host profile [45]. There is a large body of literature that has suggested that an alteration of the expression profile of HCMV-induced miRNAs may facilitate the disease diagnosis [46,47]. Even more crucial was the discovery that demonstrated the existence of HCMV miRNAs in serum and plasma. Indeed, their changes in these two environments are markedly related to hypertension, hepatitis B, and hepatitis C [48,49,50].

### 3.1. Human-Cytomegalovirus-Encoded MicroRNAs in Acute Aortic Syndrome

Given the representation in virology, human cytomegalovirus is included in the family Herpesviridae and in the subfamily Beta-Herpesviridae. The structure of HCMV is characterized by a double-stranded linear DNA covered by an icosahedral capsid. The latter is surrounded by a lipid bilayer that hosts viral glycoproteins [51]. The genome size of HCMV is the largest among human herpesviruses, reaching about 230 kb. Grey and colleagues [52] observed that the HCMV genome expresses a vast range of genes encoding small RNA molecules such as microRNAs (miRNAs) whose role is crucial in the manipulation of the host cell microenvironment and defense responses. In patients with an intact immune system, these host–virus immune integration mechanisms allow HCMV to persist throughout the lifespan. Exceptions not free from clinical problems concern HCMV infection in transplant recipients. In this cohort of patients, infection can lead to increased morbidity and mortality, thereby affecting the survival of the transplant recipient [53]. Where immunosuppression, which is a common condition in tissue transplant recipients and HIV-infected patients, is documented, prevention of HCMV infection remains a crucial goal to ensure survival and limit comorbidity.

Approximately 22 nucleotides form miRNAs, which are short non-coding RNA molecules with the function of regulating post-transcriptional gene expression. They interact with the 3′-untranslated region (3′-UTR) of mRNAs, leading to the downregulation of their expression or acceleration of their degradation [43,54,55,56]. HCMV promotes the coding of several miRNAs that are upregulated during HCMV infection [57,58,59,60]. The induced miRNA upregulation from HCMV may suggest a substantial role of these micro-molecules in viral pathogenesis. Recently, Cheng and colleagues [61] found that the HCMV miRNA levels in plasma differed in patients with TAAD as compared to healthy controls. This evidence led to a further understanding of the correlation between HCMV infection and TAAD, suggesting a well-defined role of miRNAs in the diagnosis and etiology of TAAD in the context of HCMV latent infection.

### 3.2. To Deploy HCMV Latent Infection and Promote Specific Pathological Process via miRNA

HCMV does not remain inactive in fibroblasts, and observations in endothelial cell (EC) cultures have shown a mainly focal spread. This is attributed to the characteristics of the virus discharged from the fibroblasts and EC [62]. The fibroblasts produced viruses that could infect both fibroblasts and EC. HCMV replication in human fibroblast associated with different miRNA expressions has been established in several reference papers [63,64,65,66,67]. The enhanced synthesis of non-coding RNA molecules is almost certainly driven by a coupling effect between the viral infectious process and the host genomic response. Although several studies have substantially argued that latent HCMV infection associated with reduced viral genome activity is mediated by miRNA overexpression, no convincing evidence has been reported on the phenomenon triggering inflammation in the aortic wall with progressive evolution toward AAS. Goodrum and colleagues [68] noted that in infected patients, the peculiar characteristic of HCMV is the permanence of the viral particles integrated within the host tissues for the whole duration of life in a state of dormancy. In these individuals, the absence of symptoms associated with a low level or absence of viremia has also been observed [68]. However, in this condition, the reactivation of the latent HCMV infection cannot be excluded, which, once developed, can induce marked morbidity and mortality in specific cohorts of individuals. Those most exposed to viral reactivation are immunosuppressed individuals such as tissue transplant recipients, AIDS patients, leukemia patients, and pregnant women (during the first trimester) [69,70,71,72]. Likewise, a reactivation of latent HCMV infection embodied in the aortic wall may trigger an inflammatory process, which, in hypertensive patients, can accelerate the evolution toward an acute aortic syndrome. At present, the concerns related to the treatment of latent HCMV infection are unresolved because all current prescribed anti-HCMV therapies are designed to act against the virus during replication [73].

HCMV latency is ensured in primary cellular sites, which are identified in CD34+ hematopoietic progenitor cells and CD14+ monocytes. In these cell lines, the viral genome is conserved in a non-replicative or low-copy form until the stage of conditions conducive to replication is deployed [68,74,75,76]. A precise mechanism of interaction between HCMV genes and host cell genes governs the complex process of viral latency by creating microenvironments conducive to the silent permanence of HCMV in host cells [77,78]. HCMV miRNAs participate in this sophisticated mechanism and are increasingly suggested as micro-molecules orchestrating the complex interaction between viruses and host cells [79,80]. In several former studies, the expression of HCMV-encoded miRNAs was demonstrated in HCMV-infected fibroblasts. Therefore, fibroblasts represented the first reservoir cell line of miRNA induced HCMV, because no other cell line or animal model was found to be appropriate and able to optimally study the latency of HCMV [59,60,81].

Meshesha and colleagues [82] recently found that the expression of HCMV miRNAs was detectable in monocytes and peripheral blood mononuclear cells (PBMCs), collected from subjects who experienced latent HCMV infection. Another cell line represented by THP-1 monocytic cells provided similar evidence of expressing miRNA during latent HCMV infection. The salient finding proves that functional analysis of these non-coding RNAs promotes regulatory function across a variety of viral and host genes. This role of miRNAs contributes substantially to the regulation of viral replication and innate and adaptive immune responses [79,83,84]. Therefore, more in-depth studies directed at a better understanding of the molecular mechanisms underlying HCMV latency orchestrated by HCMV miRNAs are crucial. From this context, for example, evolutionary pathological processes such as aortic dissection or the future design of therapies capable of targeting the virus during latent infection can derive greater benefit. Progression of other, often-fatal diseases such as AAS may be limited due to comorbid effects associated with viral latency.

### 3.3. HCMV-Encoded miRNAs Inhibit Viral DNA Replication

HCMV can promote latent infection by enacting mechanisms leading to the reduction in viral DNA replication. HCMV-encoded miRNAs dictate a key function in regulating viral DNA synthesis. Gray and colleagues [58] in a previous pivotal study reported the early expression of miR-UL112-1 during acute infection of human fibroblast cells. Furthermore, miRNA-UL112-1 levels were increased in subjects with sustained infection. Likewise, molecular studies performed on monocytes isolated from patients with latent HCMV infection have shown a higher expression of miRNA-UL112-1. These findings were corroborated in an in vitro THP-1 monocyte cell model affected by latent HCMV infection [82], thereby demonstrating that miRNA-UL112-1 can mediate the regulation of viral latency. The effect of upregulating miRNA-UL112-1 expression induces a decrease in viral DNA multiplication through direct downregulation of gene expression. Several independent studies have suggested that expression of the immediate early viral gene-72 (IE72) (Table 1) [81,85,86,87], is involved in the regulation of virus replication. Lau and colleagues [87] observed that in wild-type HCMV strains, deletion of the miRNA-UL112-1 target site significantly promoted IE72 expression during latent monocyte infection compared to cells in which overt infection was documented. Among other things, in a previous study, the ectopic expression of miR-UL112-1 in human embryonic kidney 293 (HEK239) cells led to the suppression of DNA replication through a direct interaction mediated by the blockade of the expression of viral IE1 (Table 1) [88].

Other miRNAs have aroused interest such as miRNA-US25-1 and miRNA-US25-2, which were expressed early during the infection with a storage that increased following the prolongation of the infection [58,81,82,89]. Meshesha and colleagues [82] demonstrated the upregulation of these miRNAs during latent-HCMV infection in monocytic cells in both the in vivo and ex vivo latency models. It was found that after the forced expression of miRNA-US25-1 and miRNA-US25-2 in human foreskin fibroblasts (HFFs), a blockade of the expression of IE72 and of the viral tegument protein pp65 was recorded. The effect was a significant reduction in viral DNA levels (Table 1) [81]. Pavelin and colleagues [89] have intensified studies on miR-US25-1 proving the attenuation of HCMV replication in primary human fibroblast cells through increased expression of miR-US25-1. The effect was a depression of the endosomal acidification process induced by the downregulation of the expression of the ATP6V0C gene (Table 1), which encodes the vacuolar ATPase (V-ATPase). The action of miR-US25-1-5p is multifactorial. miR-US25-1-5p is primarily ectopically expressed in MRC-5 cells, causing a marked decrease in viral DNA replication. Second, the expression of MiR-US25-1-5p can sustain substantial repression of the expression of multiple host genes, including tyrosine 3-monooxygenase/tryptophan 5-monooxygenase activation protein epsilon (YWHAE), ubiquitin B (UBB), phosphoprotein B23 (NPM1), and heat shock protein 90 kDa alpha, class A member 1 (HSP90AA1) (Table 1). These three proteins are implicated with a critical role in cellular bioactivity with a primary effect in conditioning the replication of the viral genome [80]. The intervention of miR-US25-2-5p is also expressed in the host. In fact, its overexpression has been observed in MRC-5 cells, leading to a decrease in the synthesis of HCMV and host genomic DNA. Qi and colleagues [90] suggested that miR-US25-2-5p may reduce HCMV genome synthesis by directly involving and suppressing the expression of eukaryotic translation initiation factor 4A1 (eIF4A1).

Shen and colleagues [91] reported an upregulation for HCMV-encoded miRNA-US5-1 in lytic infection of human embryonic lung fibroblasts (HEL). Instead, miRNA-US5-1 was poorly induced during the late period of HCMV infection involving the undifferentiated THP-1 macrophage. It is important to underline that its upregulation has been recorded in monocytes obtained from HCMV-seropositive patients [82]. Recently, the forced expression of miR-US5-1 was observed in U373 cells. The study was performed on HCMV-infected human glioma (U373) cells and showed that miRNA-US5-1 expression was strongly induced at different time points. The over-expression of miR-US5-1 in U373 cells promoted suppression of viral DNA replication through direct action driving an expression of the host DNA replication inhibitor Geminin gene (Table 1) [92].

The expression of miRNA-US33-5p was studied in HCMV-infected undifferentiated THP macrophages compared to HCMV-infected differentiated THP-1 cells, suggesting a substantial upregulation of miRNA-US33-5p in both cell lines [91]. In addition, miRNA-US33-5p expression was noted to fluctuate significantly and was lower in the plasma of healthy individuals as compared to the higher levels reported in diseased patients [93]. The oscillation of miRNA-US33-5p plasma levels taken together offers a crucial explanation for the pathogenesis of overt HCMV infection and/or HCMV latency. In fact, in cases of miR-US33-5p overexpression, the resulting effect is the inhibition of DNA synthesis and HCMV replication in MRC-5 cells. Instinctively, the action of miR-US33-5p induces downregulation of host cell syntaxin3 (STX3) expression (Table 1) [94], which is crucial for the regulation of cell growth and cytokinesis [95].

Pan and colleagues [96] reported a significant expression of miRNA-UL148D in tissues obtained from patients affected by oral lichen planus, while overexpression was not documented in patients free from HCMV infection. A marked increased upregulation associated with the accumulation of miRNA-UL14D was also observed in latently infected primary human CD34+ progenitor cells (CD34+ HPC) and myeloblastic leukemia cells (Kasumi-3). Significant evidence emerged from the study of HCMV mutant strain miRNA-UL148D that were able to replicate in Kasumi-3 and CD34+ HPC cells. A marked increase in the number of copies of the synthesized viral DNA associated with higher levels of IE-1 expression was observed in these cell lines compared to cells infected with the wild-type strain. Thus, the crucial function of miRNA-UL148D is to induce viral latency in Kasumi-3 cells by affecting the genome by downregulating the expression of the immediate-early cellular response 5 (IER5) gene. The latter favors an upregulation of cell division cycle protein 25B (CDC25B) expression, which in turn abolishes the expression of viral IE1 through the activation of cyclin-dependent kinase-1 (CDK-1). The salient finding emerging from the overall evaluation above reveals that HCMV-encoded miRNAs work to maintain viral latency with a targeted effect on both viruses and host genes, which are involved in the regulation of viral DNA replication. This process deserves to be thoroughly investigated in those pathologies that are associated with miRNA dysregulation.

**Table 1:** HCMV miRNAs blocking viral DNA replication by targeting multiple viral and host regulatory genes. Abbreviations: HFF, human foreskin fibroblast cell; HFs, human fibroblast cells; IE72, viral early immediate gene-72; pp65, viral tegument protein, viral oxygenase/tryptophan 5-monooxygenase activation protein epsilon; UBB, ubiquitin B; NPM1, phosphoprotein B23; HSP90AA1, heat shock protein 90 kDa alpha, class A member-1; eIF4A1, eukaryotic translation initiation factor 4A1; IER5, cellular immediate-early response gene-5.

MiRNA Type	Target Gene	Annotated Function	Reference Type Cells	Authors
UL112 UL112	IE72 IE1	Regulation of viral DNA synthesis Regulation of DNA synthesis	HFs HEK239	Grey et al. [85] Murphy et al. [88]
US25-2	IE72 and pp65	Regulation of viral DNA synthesis	HFF	Stern-Ginossar et al. [81]
US25-1		Regulation of viral DNA synthesis; V-ATPase induces acidification of endosome; regulation of cellular bioactivity	HFF HFs MRC-5	Stern-Ginossar et al. [81] Pavelin et al. [89] Jiang et al. [80]
US33-5p	STX3	Regulation of cell growth and cytokinesis	MRC-5	Guo et al. [94]
US25-2-5p	eIF4A1	Regulation of gene expression	MRC-5	Qi et al. [90]
US5-1	Geminin	host DNA replication inhibitor	U373	Jiang et al. [92]
ULD148D	IER5	Regulation of cell division	Kasum-3	Pan et al. [96]

## 4. HCMV-Encoded miRNAs Modulate Natural Killer and Cytotoxic T Cell Responses

We do not have robust evidence demonstrating a possible interference of latent HCMV infection with structural changes in the aortic wall. However, robust results have emerged between HCMV infection and significant impairment of the functional coupling of NK cells with HCMV-encoded miRNAs such as miR-UL112, miRNA-US25–2-3p, and miRNA-US4-1 [81,97,98,99,100]. In addition, the proteolytic shedding of NK group receptor 2D (NKG2D) ligands can not only be considered a strategy used by tumors to modulate immune recognition by NK cells and cytotoxic T cells, but an active mechanism in the regulation of inflammatory processes involved in the breakdown of the extracellular matrix in AAS and thoracic aortic dissection. The action of a wide range of metalloproteases has been evaluated with particular attention to those of the A disintegrin and metalloprotease (ADAM) family. The latter is implicated in mediating the cleavage of the NKG2D ligand and this process can be modulated by the expression of the thiol isomerase ERp5. It has been suggested that the manipulation of cytotoxic T cell (CTL) and natural killer (NK) cell responses by HCMV could be an effective strategy to mediate latency [99,100].

**Figure 3:**
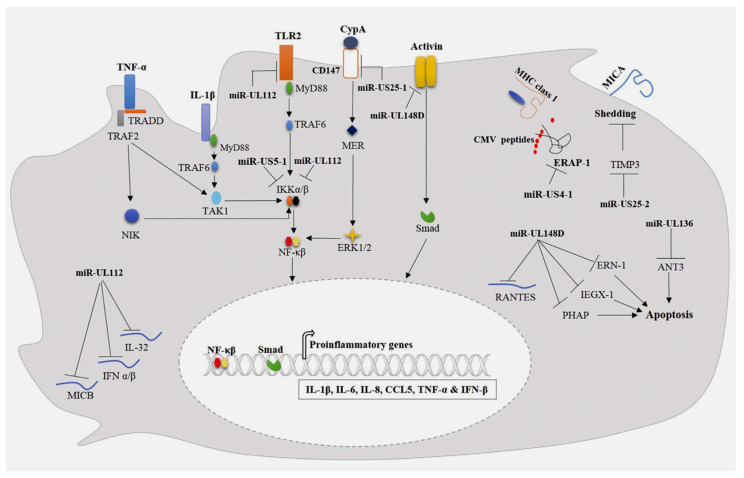
The mechanisms by which cytomegalovirus miRNAs radically change the host immune responses are depicted. The secretion of proinflammatory cytokines is inhibited by miRNA-UL112, which interferes with TLR2 by blocking IL-1β and TNF-α. MiRNA-UL112 induces activation of NF-κB signaling through downregulation of IKKα/β expression. A second action can directly target and block the expression of IL-32, IFN α/β, and MICB, which are directly blocked. The production of proinflammatory cytokines can be induced with a direct action on CD147 by miRNA-U25-1. Furthermore, the secretion of proinflammatory cytokines can be abolished through the direct interference of miRNA-U25-1 with IL-1β and TNF-α, which promotes the activation of NF-κB signaling through the revocation of IKKα/β expression. MiR-UL148D can downregulate the expression of IL-6 and RANTES by interfering with activin receptor type 1B and RANTES mRNA, respectively. Furthermore, cell apoptosis is inhibited by miR-UL148D, which interacts with IEX-1, ERN-1, and PHAP. MiRNA-UL136 can also hinder apoptosis by targeting ANT3 mRNA. The action of MiR-US4-1 is directed on ERAP-1 with an interference mechanism that affects the presentation of viral antigen through MHC class I molecules. MiR-US25-2 can increase MICA loss by targeting TIMP3, Refs. [19,20,29,61,107,108,109,110,111,112,113,114,115]. *From Abdalla AE et al. Infect Genet Evol. 2020 Mar; 78:104119*.

A non-protective effect exerted by HCMV miR-UL112 has already been demonstrated on colon carcinoma (RKO) cells, in which colon carcinoma (RKO) cells consistently expressing HCMV miR-UL112 were less subjected to aggression and killed by NK cells as compared with cells expressing the miRNA control. In detail, the mechanism involved in this process depends on the expression of miRNA-UL112, which can reverse major histocompatibility complex (MHC) class 1 chain B gene (MICB) expression (Figure 3) [81]. This ligand has the function of stress-induced NK cell activating receptor group 2D (NKG2D), which is required for NK cells to recognize virally infected cells [101]. Among other actions promoted by miR-UL112, the attenuation of NK-cell-mediated cytotoxicity was observed. The compelled expression of miR-UL112 is devoted to the function of PBMCs (lymphocytes (T cells, B cells, and NK cells), monocytes, and dendritic cells), thereby advocating the downregulation of type I interferon (IFN) expression (Figure 1). This results in a reduced expression of the lytic granule membrane protein (CD107a) on NK cells [102].

HCMV interferes with gene A(MICA) and the expression of HCMV-encoded miRNAs regulates the level of metalloprotease. The shedding of gene A (MICA) can be sustained through HCMV infection. MICA works in association with the MHC class I chain both in ex vivo infection and in patients with active HCMV infection. Specifically, a key role was played by HCMV miRNAs with a modulatory function of tissue inhibitor metalloprotease-3 (TIMP-3) expression. Esteso and colleagues [97] observed an increased shedding of the NKG2D ligand MICA post infection related to several strains of HCMV and due to an enhanced activity of ADAM17 (TNF-α converting enzyme) and matrix metalloprotease 14, advocated by a critical decrease in the expression of the endogenous inhibitor of metalloprotease tissue inhibitors of metalloproteinase TIMP3. The decrease in TIMP3 expression was associated with the increase in expression of a cellular miRNA-US25–2-3p that has been shown to target TIMP3. Furthermore, the authors identified an HCMV-encoded miRNA-US25–2-3p capable of modulating TIMP3 expression. Ultimately, the downregulation of TIMP-3 expression was correlated with an increase in miRNA-US25–2-3p expression in U373 cells (Figure 3) [97].

The role of MiR-US4-1 has been proven in the regulation of the immune response of cytotoxic T lymphocytes (CTLs). Downregulation of endoplasmic reticulum aminopeptidase-1 (ERAP1) expression is induced by the transfection of miRNA-US4-1 into U373-determined cells. This process leads to the blockade of mature viral epitopes presented in MHC class I and, consequently, to the inhibition of CTL immune responses toward HCMV-infected cells (Figure 3) [98].

Given the function expressed by HCMV-encoded miRNAs specifically to the role exerted by miRNA-UL112, miRNA-US25-3p, and miRNA-US4-1, we learned that they are modulators of CTL- and NK-cell-mediated immune recognition and the killing of HCMV infected cells. Thus, the modulation of these miRNA expressions could be crucial for the eradication of latent HCMV infection but also for preventing vascular-wall-damaging inflammatory processes mediated by metalloproteases potentially sustained by a latent HCMV infection.

Results from reports assessing the function of HCMV-encoded miRNAs may become available for the development of a new viral strategy in many fields of application. These novel therapeutic applications may work to influence the shedding of cell surface molecules involved in the modulation of the immune response. For example, Esteso and colleagues [97] assisted in explaining findings from previous reports documenting increased levels of several ADAM17 substrates in the serum of patients with CMV disease and thoracic aortic aneurysm. Consistent with these hypotheses, it has been observed that soluble MICA in the serum of transplant recipients with HCMV disease can be detected. On the other end, these data suggested that it might be worth conducting prospective studies of the activity of ADAM17, ADAMTS disintegrin, and Endothelial Fibulin-4 in a larger group of patients to evaluate whether these molecules could be a useful biomarker to identify the patients at risk of developing HCMV disease, AAS, and TAA [103,104,105,106]. In this regard, Shen and colleagues [103] highlighted distinct cell-specific functions of ADAM17 in TAA progression, promoting pathological remodeling of smooth muscle cells (SMCs; *Adam17^f/f^/Sm22^Cre/+^*) or endothelial cells (*Adam17^f/f^/Tie2^Cre/+^*) and compromising the integrity of the intimal endothelial cell barrier. ADAM17 inhibition prevented progression of aneurysmal growth, and the dual impact of ADAM17 deficiency (or inhibition) in protecting two major cell types in the aortic wall highlights the unique position of this proteinase as a potential critical therapeutic target for TAA.

## 5. Aortic Acute Syndrome and Inflammatory Response: The Role of HCMV-Encoded miRNAs in Downregulation of Inflammatory Responses

Pending more robust evidence between latent HCMV infection and the development of an inflammatory response in the AAS that may evoke thoracic aortic dissection, there is currently a substantial body of circumstantial evidence supporting the HCMV miRNA profile abnormally expressed in patients with AD [61]. Cheng and colleagues [61] noted that plasma levels of HCMV miRNAs differ in peak concentrations in AD patients and healthy controls. Using quantitative reverse-transcription–polymerase chain reaction (qRT-PCR), the expression profile of 25 HCMV miRNAs in plasma was evaluated in 20 AAS patients and 20 healthy controls. Then, abnormally expressed HCMV miRNAs were checked in a validation set of 12 AD patients and 12 healthy controls. Furthermore, HCMV infection was detected in the third cohort consisting of 20 AD patients and 20 healthy controls. Five types of miRNAs need further evaluation. The expressions of these five HCMV miRNA types were markedly different in the proportion of individuals studied, demonstrating elevated levels of these five HCMV miRNA types. In the validation set, a significant difference was observed only in the proportion of individuals with high levels of HCMV-miRNA-UL112-5p and HCMV-miRNA-UL22A-5p, two of the five HCMV miRNAs obtained in the preliminary screening. In the third cohort examined, no significant differences in HCMV DNA levels and anti-HCMV IgG concentrations were reported between AAS patients and healthy controls [61].

Inflammatory responses are crucial mediators of the reactivation of latent HCMV infection, as noted in the 1970s [68]. HCMV miRNAs can subvert host immune responses to maintain a latent infection. This process has recently been rediscovered and it is not excluded that it may involve patients who have AAS. Concerns about inflammation causing aortic wall rupture seem to have increased after the demonstration of progressive morpho-functional remodeling of the aortic wall toward a disruption profile of vascular wall layers after HCMV infection via metalloprotease alteration [19,20,29].

The TLR-2 receptor serves for pathogen recognition (PRR) and is active in several cell types, including immune and non-immune cells. TLR-2 has a crucial role in regulating mechanisms of inflammatory reactions against microbial infections [107]. Experiments performed on TLR-2 knockdown mice compared to wild-type mice demonstrated that the former were highly susceptible to CMV infection and showed a compromise in the early control of viral growth compared to the latter. Again, a marked reduction in proinflammatory secretions and a decrease in the NK cell population were determined by the deletion of TLR-2 [108]. An interference in the inflammatory response was observed by evaluating the levels of miRNAs that affect the function of TLR-2. Given this context, TLR-2 protein expression was reduced by HCMV infection of normal human dermal fibroblasts (NHDFs) and THP-1 monocytic cells, demonstrating a direct correlation and with an increased accumulation of miR-UL112-3p. Further evidence confirmed that miR-UL112-3p could directly target and downregulate TLR-2 expression in both NHDF and THP-1 monocytes. miR-UL112-3p binds TLR-2 by inducing a blockade of the activation of interleukin receptor-associated kinase-1 (IRAK1) and its downstream NF-κB signaling, thus promoting a significant reduction in the expression of proinflammatory cytokines including interleukin-1 beta (IL-1β), IL-6, and IL-8 upon activation of TLR-2-agonist-differentiated THP-1 cells (Figure 3) [109]. Similarly, the production of proinflammatory cytokines such as IL-6 and CCL5 was also reported to be induced by miR-UL112-3p and miR-US5–1 interacting with multiple cells including NHDF, THP-1 macrophages, and endothelial cell human aortic veins (HAEC). This mechanism was supported by IL-1β and tumor necrosis factor-alpha (TNF-α) production. These miRNAs knock out NF-κB signaling by promoting a downregulation of the expression of the IκB kinase complex (IKK) components IKKα and IKKβ (Figure 3) [110].

During the inflammatory process that underpins AAS, CD147/EMMPRIN is crucial for regulating proinflammatory cytokine production and T-cell activation and proliferation. CD147/EMMPRIN is a member of the transmembrane glycoprotein of the immunoglobulin superfamily [111]. It was shown that CD147 knockout could suppress the viral latency activity of HCMV integrated in the aortic wall by inducing NF-κB activation and secretion of proinflammatory cytokines such as IL-6, TNF-α, and IFN-β. It has been shown that CD147 knockout could suppress HCMV by inducing the activation of NF-κB and the secretion of proinflammatory cytokines such as IL-6, TNF-α, and IFN-β. This phenomenon could explain the onset of wall damage in patients experiencing AAS or TAA who disclose a latent viral HCMV infection. It has been shown that upregulation of miRNA-US25-15p can target and block CD147 mRNA expression, promoting the decrease in proinflammatory cytokine production induced by miRNA-US25-15p expressing HFF in the early response to infection by HCMV (Figure 3) [112]. These findings are confirmed in the study by Shen and colleagues [103] using the immunofluorescent staining method. Consistent levels of proinflammatory cytokines (interferon-γ, TNFα (tumor necrosis factor-α), interleukin-1α (IL1α), IL-6, and IL-12) and chemokines (C-X-C motif chemokine ligand (CXCL)-9, CXCL10, and C-C motif chemokine ligand-2) were elevated in the aneurysmal aorta of *Adam17^f/f^* mice but markedly suppressed in *Adam17^f/f^/Sm22^Cre/+^* and *Adam17^f/f^/Tie2^Cre/+^* mice at 3 and 14 days post TAA. Lau and colleagues [113] demonstrated that miRNA-UL148D can block IL-6 production by downregulating activin-A receptor expression (Figure 3). The latter is a member of the transforming growth factor-β superfamily that dictates a pivotal function in the inflammatory response [114]. Consistent with these findings, Lau and colleagues [113] asserted that activin-A stimulation of monocytes infected with HCMV-deleted miR-UL148D generated a significant amount of IL-6 compared to cells infected with wild-type. In another study conducted by Kim and colleagues [115], it was also proved that miR-UL148D escapes the inflammatory responses of the host through the inhibition of the recruitment of mononuclear cells to the site of infection. MiRNA-UL148D can directly drive the inflammatory process by mediating the degradation of RANTES mRNA expression (activation-regulated, normal T-cell, and secreted expression) (Figure 3). All this evidence suggests that HCMV-encoded miRNAs can reduce inflammatory responses to promote persistent infection and potentially aortic wall damage.

## 6. Future Direction

### 6.1. Implementing Knowledge on HCMV, Long Noncoding RNAs, and Aortic Dissection

Studies on long noncoding RNA (lncRNA) that constitute a class of noncoding RNAs greater than 200 nucleotides in length deserve further attention [116]. The role of these molecules is considered central to numerous cellular processes, including cell cycle, differentiation and metabolism, as well as in the development and progression of diseases including AD [117,118,119,120,121,122,123]. Several reports revealed that HCMV expresses the long non-coding RNAs (lncRNAs) RNA1.2, RNA2.7, RNA4.9, and RNA5.0. However, scant evidence emerges on the function of these lncRNAs in the virus life cycle, which may lead to latent infection and dysregulation of the host-immune-inflammatory response, as mentioned earlier in this analysis. Recently, the functional and molecular landscape of HCMV lncRNAs have been interrogated. Lee and colleagues [117] suggested that HCMV lncRNAs occupy 30% and 50–60% of the total and poly(A)+ viral transcriptome, respectively, during the life cycle of the virus. RNA1.2, RNA2.7, and RNA4.9 not only represent the three abundantly expressed lncRNAs but appear to be essential in all infection states. It has been observed that among these three lncRNAs, if depletion of RNA2.7 and RNA4.9 occurs, a greater defect in maintaining the latent reservoir and promoting lytic replication may be caused. Furthermore, researchers delineated the global post-transcriptional nature of HCMV lncRNAs through direct nanopore RNA sequencing and interactome analysis. They recorded that lncRNAs were modified with N6-methyladenosine (m6A), thus being able to interact with m6A readers in all infection states.

Li et al. studied lncRNA expression in patients with thoracic aortic dissection [118]. The study, through the extraction of aortic tissue samples, described the detection of 765 lncRNAs and 619 genes with differential mRNA expression through sequencing (fold change > 2:0, *p* < 0:01). Gene ontology analysis was evaluated by recording that lncRNAs with upregulated expression were associated with cell differentiation, homeostasis, growth, and proliferation. A smaller number of 16 lncRNAs were guaranteed with the increase in the fold change and the discovery that these lncRNAs were associated with protein-coding genes. Meanwhile, the study confirmed, using the RT-qPCR method, that P2RX7 lncRNAs, (HIF)-1A-AS2, AX746823, RP11-69I8.3, and RP11-536K7.5 were specifically correlated with P2RX7, cyclin-dependent kinase inhibitor 2B, HIF-1A, runt-related transcription factor 1, tissue growth factor connective, and the interleukin 2 mRNA receptor chain. Instead, these lncRNAs were associated with nuclear receptor activation, nuclear transcription, connective tissue development, and inflammation.

The previous study paved the way for the investigation by Sun and colleagues [119] who examined the expression profiles of lncRNA in tissues from patients with aortic dissection. Unlike the former study, Sun and colleagues isolated 269 lncRNAs and 2255 mRNAs using the high-throughput sequencing method. Analysis of the lncRNA–miRNA–mRNA network revealed that both XIST-upregulated lncRNA and p21 had similar miR-17-5p sequences. Furthermore, the predicted binding motifs of three upregulated lncRNAs (ENSG00000248508, ENSG00000226530, and EG00000259719) were associated with upregulated RUNX1. The two reported studies constitute a milestone on the interconnection of lncRNA and aortic dissection by suggesting the differential expression of lncRNA in tissues of patients with thoracic aortic dissection. Moreover, this evidence, offering an explanation on the relationship between differentially expressed lncRNAs and the related downstream mRNAs, has motivated additional speculative analyses for subsequent studies. Although the protocol mandated was different for the two studies, the sequencing methods used in the two evaluations were different; however, both reports investigated expression profiles without going into depth on related mechanisms [118,119].

Given the issues raised, a resolution was offered by Zhang and colleagues [120] who analyzed aortic tissues from patients with TAAD and identified lncRNA XIST. The investigators investigated the molecular mechanism through double luciferase reporters, qPCR, and Western blot experiments. They observed a double function of XIST. It regulates PTEN expression through the miR-17 sponge, which affects the proliferation and regulation of VSMCs. Overexpression of XIST causes the apoptosis and inhibition of VSMC proliferation, which can lead to the development of aortic dissection. Similarly, and timely, Li and colleagues [121] proved that downregulation of lncRNA PVT1 inhibited the survival, migration, and phenotypic transition of human aortic smooth muscle cells treated with platelet-derived growth factor-BB (PDGF-BB) by targeting miR-27b-3.

Renn and colleagues [122] observed that overexpression of the H19 lncRNA sponge miR-193b-3p promoted the regulation of VSMC function, including upregulation of its proliferation and migration. Conversely, the described effects were reversed after inhibition of H19 expression, whereby substantial evidence supports that H19 may be involved in the development of aortic dissection. Finally, Wang and colleagues [123] suggested that LINC01278 regulated ACTG2 gene expression through the miR-500b-5p sponge, regulating the phenotypic transformation, proliferation, and migration of human VSMCs.

### 6.2. Implementing Knowledge on HCMV, Circular RNAs, and Aortic Dissection

Circular RNAs (circRNAs) are a collection of transcriptionally expressed and spliced RNAs. They are made up of a considerable number of varieties, each of which covalently forms a closed loop through a cis linkage between its 5′ and 3′ ends with the presently hidden biological aim [124]. However, the unusual structure that characterizes these molecules results in a resistance of circRNAs to exonuclease, thereby supporting high stability in both the internal and external cell environment, with a half-life nearly 5-fold longer (48 vs. 10 h) than their parental mRNAs [125]. Kristensen and colleagues [124] suggested that the expression pattern of circRNA is tissue-specific and cell-specific and has no obvious correlation with host gene expression. CircRNAs are implicated in both physiological and pathological cellular processes through numerous mechanisms, including competition with linear splicing, the sponge-like action of miRNAs, the binding to mRNA-related proteins, and the regulation of gene expression at the epigenetic level [126,127,128]. Due to their high stability and powerful ability to regulate gene expression, circRNAs are placed at a higher biomolecular level in complicated biological and pathological processes. There is growing evidence that circRNAs play a key role in various diseases, such as diabetes mellitus, neurological disorders, cardiovascular disease, and cancer [124]. For example, circANRIL, a circRNA located in 9p21, has attracted interest for its role in atherosclerosis. CircANRIL is believed to be a locus involved in atherosclerosis that promotes binding to pescadillo homolog 1 (PES1) and restrains 60S preribosomal assembly in VSMCs and macrophages, leading to cellular apoptosis and aggravation of atherosclerosis [129].

Likewise, the environmental constancy and temporal and spatial specificity of circRNAs’ expression endowed its diagnostic potentiality. Recently, a crucial role has also been proposed for Circular RNAs (circRNAs) with the identification and characterization of circular RNA encoded by HCMV [130]. Although several studies have evaluated the profiles and potential functions of virus-encoded circRNAs, including human cytomegalovirus (HCMV)-encoded circular RNAs, the implications of virus-encoded circRNAs remain unclear [131,132,133]. Deng and colleagues [133] investigated the profile of HCMV-encoded circRNAs in lytic-infected human embryonic lung fibroblasts using deep RNA sequencing and bioinformatic analysis. In the study, 629 HCMV-encoded circRNAs with different expression patterns were recognized. The complete sequences and alternative splices of circUS12, circUL55, and circUL89 were verified using reverse transcriptase PCR (RT-PCR) with divergent primers followed and Sanger sequencing. The transcript of circUL89 was validated using Northern blot. The investigators observed that analyses of the HCMV-encoded circRNA–miRNA network disclosed the potential function of HCMV-encoded circRNAs during HCMV infection in human embryonic lung fibroblasts. Thus, it was possible to infer that HCMV infection resulted in abundant HCMV-associated circRNAs, and HCMV-encoded circRNAs might play an important role in favoring HCMV infection.

To date, few studies have reported a direct role of circRNAs in the pathogenesis of TAAD. An upregulation in TAAD has been suggested for circMARK3 and hsa_circRNA_101238 [134,135]. However, RNA sequencing (RNA-seq) to study circRNA profiles and identify differentially expressed circRNAs in patients with AD as a key differentiator has only recently been published [136]. In Tian and collogues reports, high-throughput RNA sequencing (RNA-Seq) was used to investigate the differentially expressed circRNAs, miRNAs, and mRNAs in human TAAD tissues (*n* = 10) compared with normal aortic tissues (*n* = 10). The results of RNA-Seq revealed that 506 circRNAs were markedly dysregulated (*p* < 0.05, false discovery rate FDR < 0.05, fold change > 2). The subsequent weighted gene correlation network analysis and the following co-expression network analysis recorded that tyrosine-protein kinase Fgr might play important roles in the occurrence and development of AAAD. According to the circRNA–miRNA–mRNA network, investigators demonstrated that the upstream regulatory molecule of Fgr is circMARK3. Finally, a receiver operating characteristic (ROC) curve was used to evaluate the diagnostic value of the serum circMARK3 as biomarkers for AAAD (cutoff value = 1.497, area under the curve = 0.9344, *p* < 0.0001, sensitivity = 90.0%, specificity = 86.7%). These results provided a preliminary landscape of circRNA expression profiles and indicated that circMARK3 was a potential biomarker for AAAD diagnosis.

Similarly, Zou and co-authors [135] evaluated the expression profile of circRNAs and investigated their potential functions in TAAD. They identified hundreds of differentially expressed circular RNAs in human TAD, including hsa_circRNA_101238, which may suppress the expression of hsa-miR-320a while increasing that of MMP9 in TAAD. Specifically, among the 8173 circRNA genes detected, 156 were significantly upregulated and 106 were downregulated in human TAD compared to healthy donors (*p* = 0.05). Quantitative real-time PCR demonstrated elevated expression levels of the upregulated hsa_circRNAs (hsa_circRNA_101238, hsa_circRNA_104634, hsa_circRNA_002271, hsa_circRNA_102771, hsa_circRNA_104349), COL1A1, and COL6A3, with reduced expression of the downregulated hsa_circRNAs (hsa_circRNA_102683, hsa_circRNA_005525, hsa_circRNA_103458, and FLNA). Gene ontology analysis showed that the parental genes were involved in multiple pathological processes, including the negative regulation of cell proliferation and organization of the extracellular matrix. The circRNA–miRNA co-expression network predicted that 33 circRNAs could interact with at least one altered target miRNA in TAD. KEGG pathway analysis found that 28 altered miRNAs were enriched in focal adhesion and vascular smooth muscle contraction. The hsa_circRNA_101238–miRNA–mRNA network showed the strongest association with hsa-miR-320a. Further analysis using both quantitative real-time PCR and Western blot techniques revealed low levels of hsa-miR-320a and increased levels of MMP9 in human TAD tissues.

**Figure 4:**
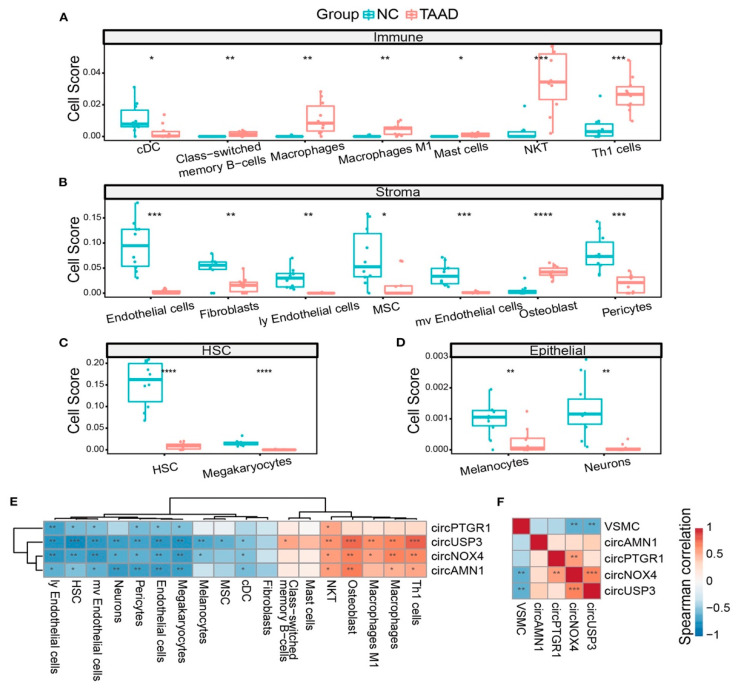
The correlation between circRNAs with the cell composition of TAAD is depicted. (**A**–**D**) TAAD aortic tissue and healthy control were assessed, and different cells, including immune cells (**A**), stromal cells (**B**), HSC (**C**), and epithelial cells (**D**), were studied. (**E**) Heatmap of Spearman’s correlation of circRNA expression and different cell scores. (**F**) Heatmap of Spearman’s correlation of circRNA expression and VSMC score. * *p* < 0.05, ** *p* < 0.01, *** *p* < 0.001, **** *p* < 0.0001 (Student’s *t*-test and Spearman’s correlation) [124,125,126,127,128,129,130,131,132,133,134,135,136,137,138]. *From Liang Q et al. Front Cardiovasc Med. 2023 Jan 13; 9:1074835*.

Most recently, Liang et al. [136] investigated the relationship among inflammation, smooth muscle dysfunction, and ECM degradation, and whether a deregulatory process was involved. The team focused on examining circRNA levels to explore TAAD from a molecular pathology perspective. Regarding four crucial circRNAs, namely circPTGR1 (chr9:114341075-114348445[−]), circNOX4 (chr11:89069012-89106660[−]), circAMN1 (chr12:31854796-31862359[−]), and circUSP3 (chr15:63845913-63855207[+]), a distinction between TAAD and control tissues was observed, indicating that these molecules represent a major disparity between the tissues in terms of gene regulation. From a functional point of view, the ceRNA network of circRNA–miRNA–mRNA predicted by the online databases, combining gene set enrichment analysis and cell component prediction, disclosed that the identified circRNAs draped all the faces of primary TAAD pathology, centralized with increasing inflammatory factors and cells, and ECM destruction and loss of vascular inherent cells along with the circRNAs. Importantly, investigators validated the high concentration and diagnostic capability of the four key circRNAs in the peripheral serum in TAAD patients. It might be speculated that viral infection leads to the release of the proinflammatory IL-32 because high levels of IL-32 were found in the serum of patients with active HCMV infection compared to control healthy individuals [137]. Although HCMV can induce IL-3 secretion during early infection of MRC-5 cells, it has been observed that during prolonged infection, IL-32 transcript as well as protein levels were decreased with increasing miR-UL112-1 expression [134]. Likewise, regarding the pathophysiological mechanism reported by Huang and colleagues, it might be pondered that ectopic expression of circmiRNA-induced miR-UL112-1 in HEK293 cells markedly reduced IL-32 production (Figure 4) [136,138].

## 7. Conclusions

While this remains a field of particular interest with promising early findings, the exact mechanisms of action should be fully comprehended to understand the complex relationship between the expression pattern of HCMV, miRNAs, circRNAs, and lncRNAs prior to expansion into routine clinical practice. The preclinical studies involving such biomarkers may one day be translated into clinical practice to allow the early detection and prognostication of outcomes and drive preventative and therapeutic options in the future.

## Data Availability

Not applicable.
